# Use of Social Media for Professional Development by Health Care Professionals: A Cross-Sectional Web-Based Survey

**DOI:** 10.2196/mededu.6232

**Published:** 2016-09-12

**Authors:** Hana Alsobayel

**Affiliations:** ^1^ Department of Rehabilitation Sciences College of Applied Medical Sciences King Saud University Riyadh Saudi Arabia

**Keywords:** social media, education, professional, health education, professional competence

## Abstract

**Background:**

Social media can be used in health care settings to enhance professional networking and education; patient communication, care, and education; public health programs; organizational promotion; and research.

**Objective:**

The aim of this study was to explore the use of social media networks for the purpose of professional development among health care professionals in Saudi Arabia using a purpose-designed Web-based survey.

**Methods:**

A cross-sectional web-based survey was undertaken. A link to the survey was posted on the investigator’s personal social media accounts including Twitter, LinkedIn, and WhatsApp.

**Results:**

A total of 231 health care professionals, who are generally social media users, participated in the study. Of these professionals, 70.6% (163/231) use social media for their professional development. The social media applications most frequently used, in the descending order, for professional development were Twitter, YouTube, Instagram, Facebook, Snapchat, and LinkedIn. The majority of respondents used social media for professional development irrespective of their age group, with the highest proportion seen in those aged 20-30 years. Social media were perceived as being most beneficial for professional development in terms of their impact on the domains of knowledge and problem solving and least helpful for enhancing clinical skills. Twitter was perceived as the most helpful type of social media for all domains listed. Respondents most frequently reported that social media were useful for professional development for the reasons of knowledge exchange and networking.

**Conclusions:**

Social media are frequently used by health care professionals in Saudi Arabia for the purposes of professional development, with Twitter most frequently used for this purpose. These findings suggest that social media networks can be powerful tools for engaging health care professionals in their professional development.

## Introduction

Social media are Internet-mediated tools that enable people to create, share, and exchange information, ideas, pictures, and videos in virtual communities and networks. Social media takes on various forms including blogs, business networks, social networks, forums, microblogs, photo and video sharing, products and services reviews, social bookmarking, social gaming, and virtual worlds. There has been a rapid growth in social media networks, with sites including Facebook, Twitter, Blogger, MySpace, YouTube, Flicker, and LinkedIn. The number of social media users worldwide has been estimated at 1.96 billion [1]. In contrast to traditional media, which are based on transmission from one source to many receivers, social media are based on transmission from many sources to many receivers.

From a health care perspective, social media can be used for a variety of reasons including enhancing professional networking and education; patient communication, care, and education; public health programs; organizational promotion; and research [2-4]. There are many benefits associated with the use of social media in health care, including increased accessibility to health information, increased peer support, and public health surveillance [4-7]. However, there are serious concerns about its use, including its governance; the accuracy, quality, and reliability of the information circulated; patient privacy and confidentiality; blurring of the personal–professional boundary; increased risk of liability; and lack of methodological rigor for social media–based research [2-8].

Health care professionals can use social media to undertake web-based professional development, connect with colleagues in their own or other professions, and keep up to date with the latest medical literature [6]. An enhanced connection with professional colleagues has been highlighted as one of the major benefits associated with the use of social media in the health care setting [6].

From an Arabic perspective, there has been an exponential growth of social media into the daily life of people, businesses, and the interaction between governments and their people [9,10]. A recent report estimated that there were more than 135 million individuals using the Internet in 22 Arab countries and more than 71 million active users of social networking technologies, with many in the health care industry in the Arab region using social media to engage with consumers and other influencers [11]. As an example, the United Arab Emirates Ministry of Health is reported as having 16,000 followers on Facebook and 9700 followers on Twitter and uses these to share its e-services, community events, health news, and health tips [11]. Similar initiatives have been commenced in other Arab countries including Saudi Arabia, Kuwait, Oman, and Qatar [11]. A study investigating the perceptions of social media users in the Arab world revealed that while social media were perceived as having many positive benefits (eg, the ability to connect with people and bring people closer together), they can also have negative effects on local culture and tradition [10].

In a recent pilot study that investigated the use of social media among health care professionals, predominantly physicians, in Saudi Arabia, Almaiman et al [12] found, in a Web-based survey of Twitter users, that 79% used Twitter to seek online health information, with users reporting that it increased their medical knowledge and improved their clinical practice. Further research investigating the use of all types of social media for professional development by health care professionals in Saudi Arabia would be useful to assess the impact of social media on supporting professional development and its perceived benefits. Therefore, the aim of this study was to examine the use of social media for professional development among health care professionals in Saudi Arabia and assess their perceptions of its benefit and impact.

## Methods

### Study Design

A cross-sectional Web-based survey was undertaken in July and August 2015. A link to the survey was posted on the investigator’s personal social media accounts including Twitter, LinkedIn, and WhatsApp. This is a pilot study that is considered an “explanatory research” [13] to investigate how health care professionals use the social media network for their professional development and what are the benefits of using social media networks for professional development as perceived by health care professionals.

### Participants

The study included a convenience sample from health care professionals in Saudi Arabia who are already using social media networks in general. Health care professionals in this study are defined as all workers holding a qualification of a health discipline working in any health care setting whether clinical (ie, health care provider) or academic (ie, education or research facilities).

### Survey

An open web-based survey was purpose-designed by the investigator to explore the use of social media for professional development among health care professionals in Saudi Arabia and assess their perceptions of its benefit and impact.

The survey comprised 3 main sections that are as follows: (1) which social media were used (options included Twitter, Instagram, YouTube, Facebook, Snapchat, and LinkedIn); (2) which social media were used, and how frequently, for professional development; and (3) the participant’s perceptions of the benefits and impacts of social media in his or her professional development. For this latter section, questions were asked about the helpfulness (categorized as “not at all helpful,” “somewhat helpful,” “very helpful,” and “extremely helpful”) of social media for professional development in terms of its benefits and impacts on 8 domains. These 8 domains were knowledge, clinical reasoning, critical thinking, clinical skills, problem solving, creativity, decision making, and patient outcome. These domains were designed based on Bloom’s Taxonomy that is a framework for classifying educational goals, objectives, and standards. The framework consists of 2 dimensions: Knowledge and Cognitive processes [14]. This section of the survey was based on the cognitive processes dimension of Bloom’s framework that consists of the following 6 categories of cognitive skills: remember, create, apply, analyze, and evaluate [14]. The survey questions explored which of these cognitive skills in relation to professional skills was improved by social media (Multimedia Appendix 1). Bloom’s framework has been used previously in studies investigating the use of social media among health specialists [15]. An additional question with set responses asked respondents to indicate their reasons for using social media for professional development. Demographic data were also collected from participants including gender, age, level of qualification, type of work (categorized as “academic only,” “clinical only,” “academic and clinical,” or “other”), country of residence, and country of origin. The first draft of this survey was reviewed by 3 health professionals similar to the target group to ensure clarity of the questions and rating scale. No major changes were suggested. A cover letter was attached to the survey explaining its purpose, the investigator’s information, the anonymity of participants, and the confidentiality of the information. No personal identification was requested or stored. The survey was distributed via URL link through Google forms.

### Statistical Analysis

Analyses were conducted using SPSS version 20.0 (SPSS Inc, Chicago, IL, USA). As well as basic descriptive data for all outcomes, results were compared between participants according to their demographic data. For the purpose of these analyses, data concerning the frequency of use were dichotomized into “yes” (“most of the time” and “all the time”) or “no” (“never” and “rarely”). Similarly, categorical data regarding the perceptions of the helpfulness of social media for professional development were dichotomized into “yes” (“somewhat helpful,” “very helpful,” or “extremely helpful”) or “no” (“not at all helpful”). Summary statistics are reported as frequency and percentages.

## Results

### Participants

The survey was sent to a total of 2500 people, which is the total number of followers of the author’s social media accounts, and 231 people responded to the survey. Among the 231 respondents, most were aged 20-40 years and the majority were female (Table 1). Level of education was evenly divided between those with, at highest, a Bachelor’s degree and those with a postgraduate degree. Most participants were involved in clinical work only or a combination of clinical and academic work. Saudi Arabia was the country of origin and residence for the majority of respondents.

**Table 1:** Demographic data for the 231 respondents.

Characteristics		Number (%)
**Age (years)**		
	20-30	111 (48.1)
	31-40	67 (29.0)
	41-50	45 (19.5)
	> 50	8 (3.5)
**Gender**		
	Female	149 (64.5)
	Male	82 (35.5)
**Highest level of qualification**		
	Bachelor’s degree or below	113 (49.0)
	Master’s degree or higher	118 (51.0)
**Type of work**		
	Academic only	51 (22.2)
	Clinical only	109 (47.4)
	Academic and clinical	52 (22.6)
	Other	19 (8.2)
**Country of residence**		
	Saudi Arabia	203 (87.9)
	United Arab Emirates	10 (4.3)
	Other	14 (6.1)
	Not specified	4 (1.7)
**Country of origin**		
	Saudi Arabia	204 (88.3)
	United Arab Emirates	9 (3.9)
	Other	11 (4.8)
	Not specified	7 (3.0)

### Use of Social Media

**Figure 1:**
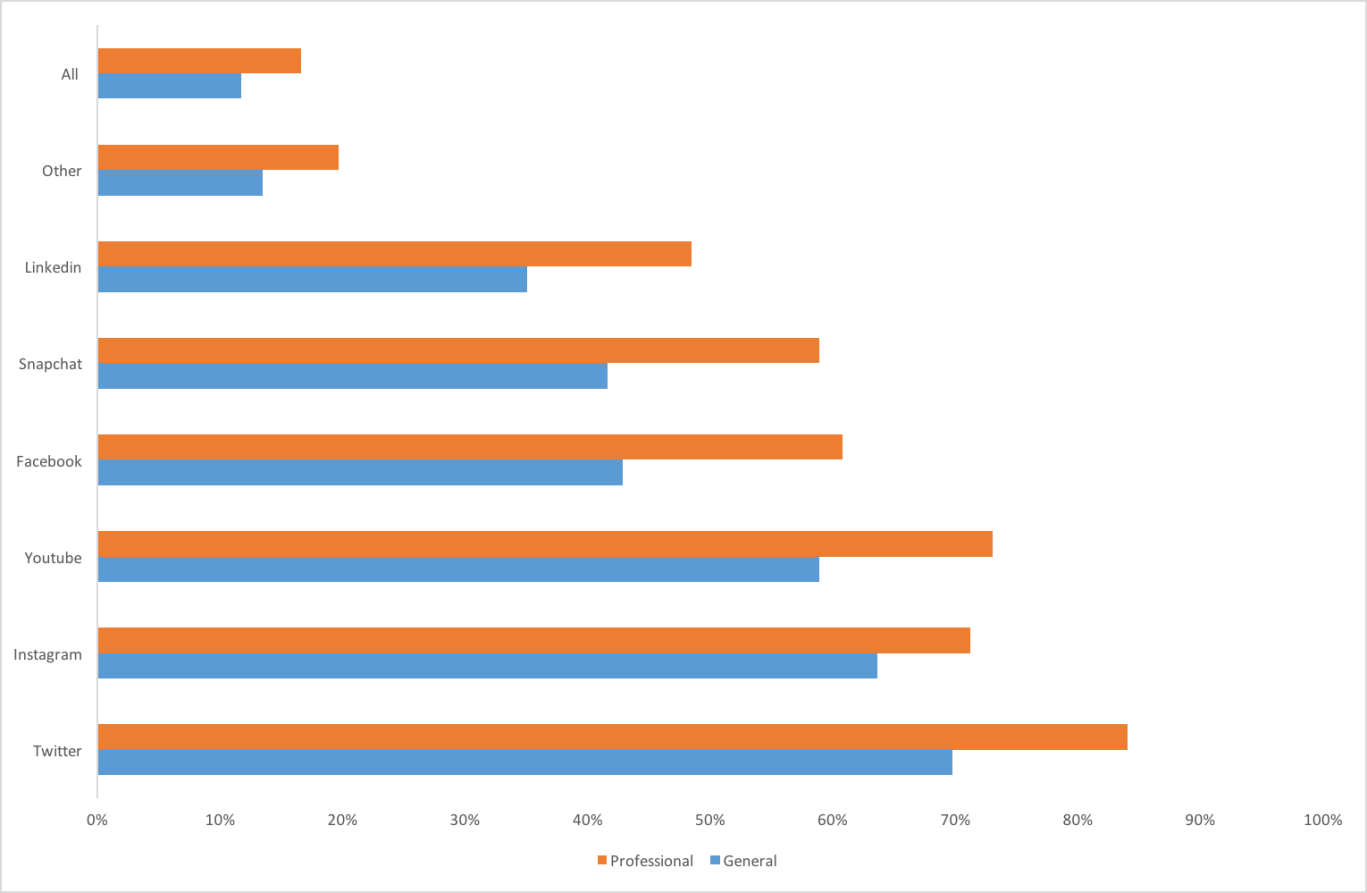
Frequency of the types of social media used generally (n=231) and for professional development (n=163).

All the 231 respondents reported that they used social media, with 163 (70.6%) reporting they used social media for professional development. The most frequently used social media platforms were similar for general usage and for professional development (Figure 1). For professional development, respondents indicated that Twitter was most frequently used (n=137; 84.1%), followed by YouTube (n=119; 73.0%), Instagram (n=116; 71.2%), Facebook (n=99; 61%), Snapchat (n=96; 60%), and LinkedIn (n=79; 49%) (Figure 1).

Among all the age groups, more than 60% reported using social media for professional development, reaching a high of 75.7% for those aged 20-30 years (Table 2). Male respondents reported using social media for professional development significantly more than females (79.3% vs 65.8%; *P*=.03). No significant association of social media usage for professional development was found with educational level or type of work.

The majority of the 163 respondents who used social media for professional development perceived social media networks to be somewhat, very, or extremely helpful in terms of their benefits and impacts across all the 8 domains listed (Table 3). Domains where social media were most frequently rated as somewhat, very, or extremely helpful were knowledge (n=161; 98.8%) followed by problem solving (n=147; 90.2%), with clinical skills least frequently rated as helpful (n=127; 77.9%). When the various types of social media were compared for their helpfulness across the 8 domains, Twitter was perceived as being most helpful across all domains (Table 4). Respondents were also asked to select their reasons for using social media networks for professional development, and as summarized in Table 5, the most frequent reasons for using social media for professional development were for knowledge exchange and networking.

**Table 2:** Use of social media for professional development according to demographic variables for the 231 respondents.

Variables		N	Used social media for professional development n (%)^a^		*P*-value
			Yes (n=163)	No (n=68)	
**Age**					
	20-30 years	111	84 (75.7)	27 (24.3)	.35
	31-40 years	67	46 (68.7)	21 (31)	
	41-50 years	45	28 (62.2)	17 (38)	
	> 50 years	8	5 (62.5)	3 (38)	
**Gender**					
	Female	149	98 (65.8)	51 (34.2)	.03^b^
	Male	82	65 (79.3)	17 (21)	
**Highest level of education**					
	Bachelor’s degree or below	111	79 (70.3)	34 (29.7)	.83
	Master’s degree or higher	120	84 (70.8)	34 (29.2)	
**Type of work**					
	Academic only	51	33 (64.7)	18 (35)	.58
	Clinical only	109	76 (69.7)	33 (31.2)	
	Academic and clinical	52	40 (76.9)	12 (23)	
	Other	19	14 (68.4)	5 (21)	

^a^Percentages are calculated relative to the total number within each row of data.

^b^Statistically significant.

**Table 3:** Perceptions about the benefits and impacts of using social media for professional development for the 163 respondents who used social media for this purpose.

Variables	Degree of helpfulness n (%)				
	Not at all helpful	Somewhat helpful	Very helpful	Extremely helpful	Helpful^a^
Knowledge	2 (1.2)	69 (42.3)	77 (47.2)	15 (9.2)	161 (98.8)
Clinical reasoning	23 (14.1)	83 (50.9)	47 (28.8)	10 (6.1)	140 (85.9)
Critical thinking	22 (13.5)	74 (45.4)	53 (32.5)	14 (8.6)	141 (86.5)
Clinical skills	36 (22.1)	71 (43.6)	48 (29.4)	8 (4.9)	127 (77.9)
Problem solving	16 (9.8)	72 (44.2)	62 (38.0)	13 (8.0)	147 (90.2)
Creativity	18 (11.0)	54 (33.1)	70 (42.9)	21 (12.9)	145 (89.0)
Decision making	24 (14.7)	75 (46.0)	55 (33.7)	9 (5.5)	139 (85.3)
Patient outcome	20 (12.2)	72 (44.2)	56 (34.4)	15 (9.2)	143 (87.7)

^a^Represents the frequency of merged responses (ie, “somewhat helpful,” “very helpful,” plus “extremely helpful”).

**Table 4:** The frequency with which the social media networks were perceived as helpful to improve professional development domains.

Variables	N^a^	Twitter	YouTube	Instagram	Snap chat	LinkedIn	Facebook
		n (%)	n (%)	n (%)	n (%)	n (%)	n (%)
Knowledge	161	136 (84.5)	118 (73.3)	115 (71.4)	81 (50.3)	75 (46.6)	39 (24.2)
Clinical reasoning	140	123 (87.9)	105 (75.0)	107 (76.4)	73 (52.1)	64 (45.7)	22 (15.7)
Critical thinking	141	124 (87.9)	105 (74.5)	105 (74.5)	72 (51.1)	64 (45.4)	27 (19.1)
Clinical skills	127	111 (87.4)	95 (74.8)	105 (82.7)	69 (54,3)	54 (42.5)	28 (22.0)
Problem solving	147	127 (86.4)	109 (74.1)	108 (73.5)	77 (52.4)	69 (46.9)	28 (19.1)
Creativity	145	125 (86.2)	107 (73.8)	107 (73.8)	75 (51.7)	69 (47.6)	40 (27.6)
Decision making	139	120 (86.3)	101 (72.7)	102 (73.4)	74 (53.2)	67 (48.2)	30 (21.6)
Patient outcome	143	122 (85.3)	106 (74.1)	107 (74.8)	77 (53.8)	65 (45.5)	31 (21.7)

^a^Represents the frequency of merged responses (ie, “somewhat helpful,” “very helpful,” plus “extremely helpful”).

**Table 5:** Reasons given by the 163 respondents^a^ for using social media networks professionally.

Reason	N (%)
Knowledge exchange	114 (69.9)
Networking	86 (52.8)
Professional development	81 (49.7)
Health promotion	70 (42.9)
New updates	66 (40.5)
Self-promotion	59 (36.2)
Employment or research opportunities	43 (26.4)
Other	2 (1.2)
All the above	43 (26.4)

^a^Respondents were able to choose more than one reason.

## Discussion

### Principal Findings

This exploratory study investigated the use of social media networks by health care professionals in Saudi Arabia for the purpose of professional development. All 231 respondents indicated that they used one or more social media networks, with 163 (70.6%) of those reporting that they used social media for their professional development. The social media platforms most often used for professional development were Twitter, YouTube, Instagram, Facebook, Snapchat, and LinkedIn. Social media were perceived as being most beneficial for professional development in terms of their impact on the domains of knowledge and problem solving and least helpful for enhancing clinical skills. Respondents most frequently reported that social media were useful for professional development for the reasons of knowledge exchange and networking.

### Comparison With Previous Work

In our sample of health care professionals in Saudi Arabia, we found that Twitter, Instagram, YouTube, and Facebook were the most frequently used social media platforms for general usage, with Twitter, YouTube, and Instagram most often used for professional development. These findings are similar to data compiled by Reyaee and Ahmed [16] who reported that Facebook, Twitter, and YouTube dominated the social media market among the general population in Saudi Arabia.

Among our sample, a higher proportion of younger age groups used social media networks for professional development purposes compared with the older age groups, although this did not achieve statistical significance. This trend may reflect that, in general, younger age groups have been reported to use the social media networks more than older age groups [17].

Using social media for professional development was perceived by the participants in this study as helpful in a number of domains, including most frequently, improving knowledge, and problem solving. These findings support those of Almaiman et al [12] who found, among physicians in Saudi Arabia using Twitter for professional development, that it was reported as being beneficial for increasing medical knowledge and in improving clinical practice.

### Limitations

One of the limitations of this study was that it was a pilot study involving only a relatively small number of health care professionals in Saudi Arabia, thus limiting the generalizability of the results. Another limitation was that as the sample was drawn from health care professionals already active online and further utilized a Web-based survey, it is likely that the sample was biased toward those who were more likely to use social media for professional development. Nevertheless, the results provide new data concerning social media usage for professional development among health care professionals in Saudi Arabia who are already engaged in online social media. Further research using offline methods of recruiting participants will be essential to confirm and extend the results of this study.

### Conclusions

We found that the majority of health care professionals in Saudi Arabia participating in this study used social media for the purposes of professional development. Twitter, YouTube, Instagram, Facebook, Snapchat, and LinkedIn were the media platforms most often used for professional development. In terms of their benefits, social media were perceived as being most helpful for professional development for improving knowledge and problem solving. These findings suggest that social media networks can be powerful tools to engage health care professionals in their professional development.

## Appendix

Multimedia Appendix 1 The survey questions.
