# Supplementary material for: Use of Social Media for Professional Development by Health Care Professionals: A Cross-Sectional Web-Based Survey
Source: JMIR Med Educ. 2016 Sep 12;2(2):e15. doi: 10.2196/mededu.6232 (PMC5053809; doi:10.2196/mededu.6232)
Supplement: Multimedia Appendix 1 [file mededu_v2i2e15_app1.pdf]

How socially connected are you?

Dear health care professional,

Thank you for taking few minutes of your time to respond to this survey about your use of social media for professional purposes. Your participation will provide an insight on the perceptions of health care professionals living in the Arab region on the use of social media for their professional development.

If you have any questions or comments, please do not hesitate to contact me using the contact information below.

Best wishes.

Hana

Hana I. Alsobayel, BSc. PT, MSc., PhD.
Assistant Professor in Physical Therapy
King Saud University
Saudi Arabia
hsobayel@ksu.edu.sa

* Required

Social media network usage

1. **Which of the following social media networks do you use? ***
   You can select more than one network.
   Check all that apply.

   - [ ] Facebook
   - [ ] Twitter
   - [ ] Instagram
   - [ ] Linkedin
   - [ ] Youtube
   - [ ] Snapchat
   - [ ] All of the above
   - [ ] Other: ____________

2. **How much time do you usually spend using social media networks on a daily basis? ***
   Mark only one oval.

   - ( ) Less than an hour
   - ( ) up to 2 hours
   - ( ) up to 3 hours
   - ( ) up to 4 hours
   - ( ) 5 hours or more

3. **Do you use social media networks for professional purposes?** *
Mark only one oval.

- ( ) Yes
- ( ) No *Skip to question 7.*

Professional use of social media networks

4. **Rate your use of social media networks for professional purposes.** *
(e.g. networking, health promotion, etc.)
Mark only one oval per row.

	Never	Rarely	Most of the time	All the time
Facebook	( )	( )	( )	( )
Twitter	( )	( )	( )	( )
Instagram	( )	( )	( )	( )
Linkedin	( )	( )	( )	( )
Youtube	( )	( )	( )	( )
Snapchat	( )	( )	( )	( )
Other (as specified in question 1)	( )	( )	( )	( )

5. **What are your reasons for using social media networks professionally?** *
You can select more than one reason.
Check all that apply.

- [ ] Networking
- [ ] Health promotion
- [ ] Knowledge exchange
- [ ] Employment/research opportunities
- [ ] Professional development
- [ ] Self promotion
- [ ] all of the above
- [ ] New update
- [ ] Other: ____________________

6. **Rate the impact of using social media networks on your professional development and practice.** *
Mark only one oval per row.

	Not at all helpful	Somewhat helpful	Very helpful	Extremely helpful
Improving knowledge about the profession				
Improving clinical reasoning skills				
Improving critical thinking skills				
Improving clinical skills				
Improving problem solving skills				
Improving creativity				
Improving clinical/professional decision making				
Improving patient outcomes				

Personal information

This will be used for research purposes only.

7. **Your age in years** *

____

8. **Your gender** *
Mark only one oval.

- Male
- Female

9. **Your nationality** *

____

10. **Country you're living in** *

____

11. **Which city?** *

____

12. **Your professional qualification** *
Check all that apply.

- [ ] Diploma
- [ ] Bachelor
- [ ] Masters
- [ ] Doctorate
- [ ] Other: ____________________

13. **Your line of work is mainly:** *
Mark only one oval.

- ( ) Academic
- ( ) Clinical
- ( ) Both
- ( ) Other: ____________

14. **Number of years of clinical experience following your entry level education:** *

____

15. **Comments**

____
____
____
____
____

16. **Name and contact information**
(Optional)

____
____
____
____
____

Powered by
Google Forms
